# Placenta Previa, with Special Reference to Treatment1Read before the tenth meeting of the American Association of Obstetricians and Gynecologists, at Niagara Falls, August 19, 1897, and published in the *American Journal of Obstetrics*.

**Published:** 1897-10

**Authors:** W. H. Wenning

**Affiliations:** Cincinnati, Ohio, Professor of obstetrics in the Woman’s Medical College; gynecologist to St. Mary’s Hospital; Fellow of the American Association of Obstetricians and Gynecologists


### PLACENTA PREVIA, WITH SPECIAL REFERENCE TO TREATMENT.[1]

By W. H. WENNING, M. D., Cincinnati, Ohio,

Professor of obstetrics in the Woman's Medical College; gynecologist to St. Mary's Hospital; Fellow of the American Association of Obstetricians and Gynecologists.

A FEW years ago I had the honor to present to this association an essay entitled A study of the pathology of placenta previa. Within it were set forth the various theories advanced up to that time on the etiology and interpretation of clinical symptoms in this most interesting anomaly of pregnancy. Its intimate connection with the doctrine of expansion of the lower segment of the uterus and retraction, as well as contraction, of the upper segment of the uterus, was discussed and an endeavor made to harmonise the various conflicting theories in reference to the anatomical position of the inner os during labor in its bearing upon the development of the previal placenta. The length of the paper precluded the more practical consideration of the treatment of this accident. A regret was then expressed by several members that I had not entered into this subject in its therapeutical aspects, which I accordingly briefly reviewed in the interesting discussion that followed the reading of my paper.

On this occasion, therefore, I have taken the liberty to recur to this subject, not so much with the intention of presenting any new and startling contribution, as to simply review (1) some of the means at our disposal for arresting the hemorrhage, the cardinal symptom of placenta previa and (2) the methods of expediting labor, which is almost always imperative in the interest of the lives of both mother and child.

In no other condition of pregnancy is the danger to life so imminent, in no other is a knowledge of the proper methods for averting this danger so essential, and in no other is a judicious discrimination as to the relative value of each of the several procedures so necessary as in placenta previa.

An affection in which the maternal mortality has been at various times computed as ranging from 25 to 50 per cent. and the fetal from 50 to 75 per cent. certainly merits our closest attention.

Fortunately for womankind, this fearful death-rate has been greatly lessened within the last few decades, thanks to improved methods of treatment and earlier recognition of this condition,

1. Read before the tenth meeting of the American Association of Obstetricians and Gynecologists, at Niagara Falls, August 19, 1897, and published in the *American Journal of Obstetrics.*

even if no very great strides forward have been made in the saving of infant lives.

Unfortunately, however, even at the present time this decreased mortality has not been so general as it should have been and it is only from well-conducted institutions, under skilful management, that successful results of a marked character have been obtained. The maternal mortality in a number of recorded cases has been decreased to the encouraging figure of from 4.50 to 7 per cent., yet the general fatality in unselected cases still reaches the high figure of from 25 to 33 per cent.

When we examine into the cause of this great difference we must, of course, make allowance for the different degrees of placenta previa, which, in many instances, may have been recognised only in the fatal cases, but certainly much better results should have been obtained in general practice if two absolute rules could be more generally inculcated—namely, (1) timely intervention and (2) a proper judgment of the means applicable to the case in question.

The relative merits and demerits of this or that method have time and again been reiterated, so that the conclusion naturally forces itself upon us that every procedure has its advantages or disadvantages and that no particular treatment is applicable to each and every case. This will also explain the fallacy of statistics, which perhaps are nowhere so unreliable when made conformable to a certain mode of action, as in placenta previa.

To illustrate I will briefly summarise the methods of treatment resorted to by fifty American writers in the same number of cases reported in the last five years, almost uniformly with a good result —at least for the mothers. Three depended solely on expectant treatment; three used the tampon alone, one with rupture of the membranes; three punctured the membranes; four instituted bipolar version; three ordinary version; four adopted Barnes's method of dilating the cervix, four resorted to this method and followed it by version; two by the use of the forceps, and two by version and forceps applied to the aftercoming head. In fifteen instances recourse was had to accouchement forcé—namely, manual dilatation of the cervix followed by version. In three instances separation of the placenta was practised and in two instances Cesarean section was made. Of this number only two deaths are recorded—one after the use of the tampon, the other following Cesarean section.

This collection, taken at random, simply proves that all methods may be followed by good results and that no fixed rule can be established for every case. It is remarkable, however, that notwithstanding the excellent results achieved after the rehabilitation of the Braxton Hicks method of bipolar version only four cases are reported as having been treated in this manner, whilst the much-condemned procedure known as accouchement forcé, viz., manual dilatation followed by version, is mentioned as often as fifteen times, and that among these last cases we find some of the best and most eminent teachers of our land.

Discarding the more antiquated forms of treatment, which by common consent have fallen into disuse, we will confine ourselves to a discussion of three of the methods most commonly employed. These are (1) the tampon, (2) rupture of the membranes and (3) version. Each of these means may be used by itself, or, which is more frequent, in combination with one or two of the others. It is my purpose to show that each method of procedure has its specific indications and that it is not just either to extol one or condemn the other, to the exclusion of the rest. It is the knowledge of the applicability of a specific treatment in a given case and a nice discrimination among the various methods that will give the best results.

While not wishing to appear dogmatic and admitting that there may be some exceptions to the following rule, I would make the broad statement that the tampon is applicable only before dilatation of the os, rupture of the membranes after the onset of labor pains, and version after sufficient dilatation or dilatability of the cervix.

It is the nonobservance of this principle that, in my estimation, is responsible for the fearful mortality that has followed each of these methods when employed injudiciously, that is, at the wrong period; and to the observance of this principle that the excellent results are attributable after judicious use of each of these three methods.

It is just as irrational to use the vaginal tampon when the os is sufficiently dilated for delivery, either spontaneous or artificial, as it would be to attempt version with a closed or rigid os. As a rule it is as improper to puncture the membranes before labor has sufficiently advanced to permit the presenting part or head to engage in the cervix and thus arrest bleeding, as it is to wait for a spontaneous rupture of the membranes when bleeding occurs from a well-dilated or dilatable os, readily permitting manual interference.

Then again, each method is subject to its modifications and such questions as the following may present themselves: When shall we use the vaginal and when the cervical tampon? Shall we rupture the membranes as soon as the os is permeable to two fingers for the performance of bipolar version, or shall we wait until the hand can be gradually introduced for ordinary podalic version? When shall we resort to Braxton Hicks's method and when to internal version?

Whilst one or two of these methods may be elective, there are proper indications for each which may now be considered in detail.

### THE TAMPON.

The vaginal tampon is simply a preparatory measure. It serves to arrest bleeding until the time comes for active interference. Its office begins with the onset of hemorrhage and ceases with dilatation of the os. It serves a double purpose in that, when properly applied, it checks bleeding and assists in bringing on labor. As both are accomplished by pressure, it is necessary that the tampon be at the same time large and firm. This property of inducing labor, which often contraindicates the tampon in abortion, serves a good purpose in placenta previa, for it is positively proven that no woman who has hemorrhage of such an alarming extent as to require active measures for arrest in placenta previa is safe until she is delivered. The indication of labor, to my mind, is imperative with the first onset of copious hemorrhage. What is more reasonable, therefore, than to check the latter and expedite the former? The tampon fulfills both indications. By its means we gain time for further manipulations, and at the same time maintain the strength of the patient.

The opponents of the vaginal tampon offer the following objections: (1) It does not check hemorrhage; (2) it is apt to produce sepsis.

The first objection is, I fear, too often well-grounded. In most instances, however, it is undoubtedly due to the form of the tampon. If constructed as is customary for abortions and ordinary tamponment of the vagina, it is worse than useless, for it gives rise to a false sense of security. In a number of consultations in which I have been called, I have not once seen the tampon employed *secundum artem.* No wonder, then, if the tampon should fall into disrepute. Unless the vagina be completely packed throughout the length and breadth of this cavity, "from dome to pit," can it offer any safeguard against hemorrhage? If such older obstetricians as Leroux and Wiegand have had excellent results, it is because of the thorough method which they employed. The relaxed condition of the vagina entails with it a capacity which is simply enormous. It is immaterial whether cotton or gauze be used for that purpose, provided the quantity be only sufficient; "a hatful" of cotton balls, as Pajot says, or 1,500 grams of gauze, as Aubard claims, being required for that purpose. My own preference is for iodoform or sterilised gauze, as it is more convenient both for introduction and removal. By its means the cavity can be rapidly filled, a matter of great importance when time is so valuable. The patient should be placed in Sims's position, in order to invoke the aid of atmospheric pressure in distending the cavity. A large Sims or other retractor is introduced and a roll of gauze, about three fingers in width, may be rapidly passed into the vagina. If possible, the cervix should first be packed and then the whole vaginal cavity tightly packed. Although usually a Sims retractor is employed for that purpose, I prefer a Neugebauer speculum, which I use for all tamponing in private practice, as its open end is particularly well adapted for this purpose; it is self-retaining when used with its fellow and is perfectly aseptic. Its simplicity and special adaptability are its most commendable features. I may be pardoned for calling attention to this rather elementary subject, but as I am speaking more for the general practitioner, upon whom this duty will first devolve than the specialist, I desire to facilitate this maneuver as much as possible. This retractor has the further advantage of not in the least displacing the solid plug when the instrument is withdrawn at the end of the operation.

It cannot be denied, however, that even with the greatest precautions we may fail occasionally of accomplishing our purpose. In some instances the blood will sicker through or alongside of the tampon, but more frequently the retraction of the uterus will draw the cervix away from the tampon and blood will be effused between these two surfaces. Internal hemorrhage is not likely to occur, as the intrauterine pressure of the fetus and tense bag of waters will exert sufficient pressure against the cervix in the early stage of labor, the only time when the vaginal plug is admissible. At any rate, constant watchfulness and observation are necessary, and *under no circumstances should a physician leave his patient from this time onward until the end of labor*.

How long should the tampon remain? As long as there is no external or constitutional evidence of hemorrhage. If no bleeding occurs it is not necessary to remove it at the end of one hour, as Barnes would have it, but neither is it safe to wait for its expulsion by the natural labor pains as Pajot advises. It should be removed at the end of about twelve hours, unless circumstances demand an earlier removal.

The danger of sepsis is averted by the known rules of asepsis and antisepsis in cleansing the genital tract and the use of surgically clean material. The blood itself is an excellent antiseptic, and if care be taken against the introduction of any contaminated object the risk of sepsis is indeed very slight.

The vaginal tampon when properly applied is therefore indicated in the following conditions: (1) marked hemorrhage in pregnancy, (2) moderate or profuse hemorrhage in the beginning of labor, and (3) when the patient can be kept under strict supervision.

When the os is sufficiently dilated for the introduction of a cervical tampon, this should be resorted to. Sponge tents, tupelo or seatangle tents, although still advocated by some, have justly been discarded on account of the danger of infection in the first instance and the liability to slip out of neck of the womb in the latter case. Preferable are the various forms of rubber appliances, as Barnes's bags, Braun's colpeurynter (originally, however, intended for the vagina, as its name implies); or the several French appliances devised by Tarnier, Chassagny, Champetier de Ribes and others. Of these, Barnes's bags are undoubtedly most ingenious, and theoretically well adapted. Practically, however, they frequently fail, for the following reasons: difficulty of introduction, a tendency to slip out notwithstanding their constricted shape, and, lastly, the necessity of early removal for the introduction of a larger size. Notwithstanding that these disadvantages have been somewhat obviated by the modifications of Cowan, Fehling, McLean and the like, they have never become very popular.

The most serious objection is, however, that they do not always sufficiently tampon the cervix to prevent leakage, as shown in the following case:

Some years ago I was summoned to a case of placenta previa by a medical friend who had endeavored for several days to check hemorrhage with the ordinary vaginal tampon. On examination I found the os sufficiently dilated for combined version. He preferred, however, the continued use of the tampon, and as a compromise I suggested

Barnes's cervical tampon. I accordingly introduced the largest size that would enter the cervix, and, when it was well filled, satisfied myself that the cervix embraced the bag tightly; not a drop apparently could leak out. As I had been called to attend another case of hemorrhage, I left the case in his charge until I could return. Unfortunately, he, too, left the patient soon after, and on my return, half an hour later, I found the patient almost exsanguinated for the loss of blood. The bag had slipped into the vagina, and I was forced to introduce my hand as far as possible to check the terrible bleeding that was taking place. With the greatest difficulty I delivered the patient by version, or accouchement forcé, before the bleeding could be stopped. Unfortunately, septicemia set in, and six weeks later the woman died with a phlegmasia alba dolens.

This case well illustrates the improper use of the tampon in the beginning, the unreliability even of cervical tamponing in advancing labor and the necessity for constant vigilance.

Over this intracervical tampon of Barnes, the supracervical bags of Tarnier, Champetier de Ribes and the like, are a decided improvement.

Braun's colpeurynter (in this instance more properly called metreurynter) has also been used by Schauta and the Vienna school for tamponing the cervix. Of all these appliances, Champetier de Ribes's bag seems to me the most efficient; it is fast finding favor, not alone with French, but also with German and English obstetricians. The conical shape of this bag adapts it most perfectly for controlling hemorrhage by pressure on the cervical portion of the lower uterine segment and also prevents its expulsion until the cervix is large enough to be followed by the advancing head or permit the introduction of the hand. Its hydrostatic pressure exactly imitates nature by opening the cervix from above downward. A further advantage is its inelasticity, by which it exerts a stronger pressure upon the surrounding tissues than Braun's colpeurynter or Tarnier's intrauterine balloon. It can be introduced when the os is dilated to two fingers' breadth. Consequently it enters into direct competition with Braxton Hicks's method of combined or bipolar version. It has the advantage over this latter procedure that it is easier of execution, requires no assistant for anesthesia and takes less time than combined version. It closely follows the method and principle of vaginal tamponment and is, therefore, more readily learned. The shape of the bag at once reminds one of the breech and thigh of the child, acting in this manner similarly to the extended limb, as is recommended in the final stage of

Braxton Hicks's method of version. It performs the same office without disturbing the axis of the child. Its disadvantages are said to be the danger of producing sepsis and the tendency to displace the presenting part and thus preventing the head from engaging in the cervix. The former can be obviated by chemical antisepsis and should not be attended with greater danger than the introduction of the hand in version. The second objection is also not tenable, for either the head may be pushed into the lower part of the uterus or the hand introduced and the delivery concluded by version. At any rate, transverse presentations are very frequent in placenta previa; even if the presentation be vertical and should the head be pushed aside, the very act is accomplished which is the first step in bipolar version.

A perusal of recent German literature, notably the reports of the proceedings of the last meeting of the German Gynecological Society at Leipsic,[2] show that such ardent supporters of the Braxton Hicks method as Hofmeier, Schatz, Kuestner and others admit the advantages of the cervical tampon and look upon it with much greater favor than vaginal tamponment.

### RUPTURE OF THE MEMBRANES.

Contrary to the accepted rule in normal obstetrical cases—to preserve the membranes until full dilatation of the os has occurred—early rupture of the bag of waters for the purpose of precipitating uterine contractions and thereby arrest hemorrhage by fetal pressure, has been very urgently advised by several eminent authorities; other equally experienced obstetricians have as stoutly disapproved of this procedure until delivery could be readily effected through a dilated or dilatable cervix. Thus Barnes says, "the puncture of the membranes is the first thing to be done in all cases of flooding before labor sufficient to cause anxiety. *It is the most generally efficacious remedy and it can always be applied.* It is sometimes sufficient in itself; it does not materially interfere with the resort to further steps."[3]

Schröder, although an advocate of the use of the tampon in the beginning, likewise advises early rupture of the membranes. He significantly adds, "there are no contraindications."[4] He lays down the following rule of treatment: "Rupture the membranes, bring down one foot, but wait with extraction."[5]

This doctrine is, however, not adhered to by all his pupils, for Veit observes: "Although rupture of the membranes may be theoretically correct, I would recommend it in practice only when, with a slight previal placenta, the os is well dilated and the size of the child will enable its immediate engagement. In doubtful cases combined version should always be preferred, but sight must not be lost of the fact that with the loss of the waters combined version becomes very difficult ; we should not attempt rupture of the membranes and then, when the hemorrhage ceases, resort to combined version."[6] Later on he says : "Rupture of the membranes alone suffices in those cases in which the os in a vertex presentation is considerably dilated and the head enters upon it."[7]

In other respects he is a firm supporter of Braxton Hicks's bipolar version.

Spiegelberg, the most pronounced apostle of accouchement forcé, after stating his objections to the method of bipolar version, that it is not always easy of execution and that it does not arrest hemorrhage, continues : "For the same reason rupture of the membranes with presentation of the vertex is in general not advisable. Experience teaches that this method is followed by a good result only in those cases in which there are labor pains and the head is soon forced into the inlet. Unfortunately, in placenta previa good pains are often wanting and rupture of the membranes must often be followed by version, now performed under more unfavorable circumstances than if it had been done at once. Better, therefore, do the whole thing."[8]

It will be seen from these quotations that the propositions are diametrically opposed to each other. The advocates of early puncture aim to excite labor pains by evacuating the liquor amnii ; the opponents claim that good pains and a well-dilated os alone justify this procedure.

In my judgment both views are extreme. It should always be our endeavor to imitate nature as much as possible, even in so anomalous a condition as placenta previa. We should first try to check hemorrhage by means of the vaginal tampon, then by the cervical plug, always, if possible, preserving the membranes until rupture, either spontaneous or artificial, can be followed by rapid delivery. Moreover, early rupture, in a certain measure, impedes bipolar version, for this operation is much more difficult to perform with the waters lost than with the membranes intact. It will be observed that advocates of early rupture are at the same time the partisans of combined version. They impede their own work. It also lessens the chance for the deflected head or breech to enter into the cervix.

On the other hand, when hemorrhage continues unabated and, in spite of good vaginal tamponing a reasonable time, labor pains do not set in, it may become necessary, in the interest of the mother, to disregard all rules and hasten delivery, not for the purpose of exciting pains, but to terminate labor as rapidly as possible, if need be, by accouchement forcé. Complete (or as it is generally, though not always correctly, called central) placenta previa will require this early puncture of the membranes more frequently than the incomplete or lateral variety, as the hemorrhage is more profuse and necessitates rapid delivery without awaiting the onset of natural pains.

As a rule, therefore, the membranes should be ruptured early in labor ; (1) when hemorrhage is profuse and cannot be checked by tamponing ; and (2) when pains are absent and it is evident that evacuation of the liquor amnii will stimulate the uterus to contraction. It is indicated late in labor ; (1) when the os is sufficiently dilated that either spontaneous delivery will soon follow ; or (2) manual or instrumental delivery can safely be resorted to.

VERSION.

Much on this subject has already been said in the discussion of the two preceding methods of procedure. It is undoubtedly the most important and most commonly practised method of hastening delivery in placenta previa. The breech and thigh of the child arrested in the cervix act as a tampon and gives us an excellent means of checking hemorrhage before extraction of the child really has taken place. No one will dispute its efficiency. The only question at issue is, can it be resorted to sufficiently early to act as a tampon and at the same time expedite delivery without danger to mother and child ? or should it be resorted to only in those cases in which other methods have already been tried which proved unavailing and rapid extraction becomes imperative ?

The *time* of performance is here an important element. At an early date no other method of version than that known as bipolar version is safe and practicable, at a latter date direct or internal version is not only possible, but much more easy of execution.

(*a*) Braxton Hicks has undoubtedly conferred a great boon upon the obstetricians of the whole world and through them upon suffering womankind, when he devised his now so widely and favorably known method of combined internal and external, or bipolar version. He has given us a method by which we can relieve women of imminent danger of death long before any other means are at our service. For no other condition in obstetric practice has this been a greater blessing than just in placenta previa. As stated in the early part of this essay, many, nay almost all, of our good results in lessening maternal mortality have been due to the skilful use of, and timely resort to combined version. It is but recently, however, that its beneficent results have been made manifest, when the Berlin school, under the guidance of Schröder, and followed by Hofmeier, Behm, Wyder and Lomer, obtained such brilliant results. To them belongs the credit of having brought this method more prominently again before the world and for having endeavored to introduce it more generally into practice. Without wishing to detract one iota from their merits, I may be pardoned for expressing a doubt whether such excellent results will ever be obtained outside of well-regulated lying-in institutions, where skilled operators and assistants, careful supervision and early observation, go hand in hand together. This combination is not apt to be found in general practice, where the merest tyro may suddenly be brought in confrontation with a desperate case, alone without assistance, and at a time when every moment is precious.

Let us briefly review under what circumstances we are advised to resort to combined or bipolar version. Barnes, Schröder, Veit and others all say: as soon as two fingers can be inserted into the os, no matter what period of pregnancy or stage of labor. In theory this is correct, but is it always possible to make any impression on the presenting part when the fetus is still so high in the pelvis? It is true that rupture of the membranes may bring on labor pains and thus force the body of the child sufficiently downward to be reached by the examining finger. But we have already seen that this loss of fluid materially impedes the rotation of the child, which must be done with ease to render the method effective. We have already stated that rupture of the membranes, unless immediately by combined version, is really a contraindication against its performance.

It is true we are told that the hand must be introduced entirely into the vagina, so that the extended finger or fingers may penetrate the long cervix.[9] But who can do this at so early a stage of labor without inflicting great pain upon the patient, not to speak of the impossibility of forcing the whole hand into the vagina?

We are again told that the patient must be thoroughly anesthetised, when it will be easy to execute all the maneuvers necessary for this operation. True, but this requires competent assistance, thorough obstetrical knowledge, both in diagnosis and treatment and such other favorable surroundings, not always obtainable in ordinary practice. Where the combination above mentioned is at hand, the results that have been attained are sufficient proof for the excellence of the method.

The reproof that infantile mortality has not been lessened, nay perhaps even increased, is to my mind no valid objection, as the life of a mother is paramount to that of a number of children, and where the maternal loss of life is one-tenth to one-fifth of that of former statistical results, no further argument is needed. It must be added, however, that the primary object aimed at—namely, cessation of hemorrhage, is not always obtained, as proven by a number of observers, among others by Runge in a well-illustrated and remarkable case.[10]

The question of slow extraction, or leaving expulsion of the child to nature, need not be discussed at any length. It is now almost the common opinion that slow extraction does not imperil the integrity of the cervix, but facilitates labor and more securely checks hemorrhage.

Finally, then, under the limitations above mentioned, we can strongly recommend this method, but should as urgently warn against it as a hazardous attempt when it cannot be readily completed, adding to the difficulties already encountered, unless skilfully executed. Under these circumstances the cervical tampon, as already described, is a less difficult and less complicated procedure.

Attention has already been called to the similarity in shape and action of Champetier de Ribes's bag and the ham of the fetus. One advantage for the latter, however, in the completion of the act of combined version is that the leg of the child offers us a means of delivery at the same time that it acts as a natural tampon, whereas the expulsion or extraction of Champetier's bag does not yet conclude labor, but must be followed by version, or, in favorable cases, by the use of the forceps, unless the head of the child immediately engages in the cervix and takes the place of the artificial tampon that preceded it.

(*b*) Internal version, by which we mean ordinary podalic version, is the oldest and best known method of terminating labor in placenta previa when the os is large enough to admit the hand.

When proper precautions against hemorrhage have been taken in the earlier or dilating period and danger has been successfully averted until the os has become dilated—or at least dilatable—podalic version is the most practical, direct and effective mode of delivery and, during the performance of the act, of checking the bleeding. The hand and arm of the operator first compress the bleeding surface ; then, when the foot and, following it, the lèg and thigh are brought down, this hemostatic pressure is continued, just as in the final stages of Braxton Hicks's method of bipolar version. There can be no objection to this method when the circumstances are as favorable as in the instance just mentioned. But a more important question is, shall we resort to direct version at an earlier stage of labor, before the os is sufficiently dilated or dilatable for the introduction of the hand ? To accomplish its purpose, the hand must force its way through a rigid and, as yet, undilated os and unrelentingly continue its pressure until it wedges its way into the uterine cavity, seizes the foot and rapidly brings it down. This is what is literally meant by accouchement forcé, or forced delivery. However, this definition has been somewhat modified and the term has been sometimes applied to a method of procedure much less brusque than might at first sight appear.

The question of propriety or impropriety of this operation will be measured entirely by the degree of force to be used and this again will be determined by the period of its attempt. The term will, therefore, admit of a very wide interpretation and will always require a qualifying explanation. When, therefore, cases are recorded as having been delivered in this manner a description of the permeability of the os is always in order, to determine whether the method was applicable in the given case or not. It is with this understanding that I proceed to express either my approval or disapproval when I at one time extol and then again condemn this method.

Early in labor, when the os is firm and unyielding, when a forcible dilatation would be apt to be attended with laceration of the cervix and possibly gangrene, when other safer means are at hand to check the bleeding and at the same time aid the natural course of labor to such a point when artificial means can be resorted to without injury to the parts, accouchement forcé is certainly to be condemned. The only justification for such a course lies in such a state of affairs that no time is to be lost, when the bleeding is so enormous and uncontrollable that the patient is sure to die unless rapidly delivered. Here the question hinges upon the point whether the woman shall be allowed to die undelivered or be given the (it may be) slender chance of recovery by forced delivery.

When, however, the cervix is not very rigid and gradually yields to the manual dilatation effected by one finger following the other until the whole hand can be introduced and then version cautiously but unhesitatingly performed, this method claims advantages over any of the others.

In the first place, if the cervix is soft but not friable, the fingers and hand form the best dilator and intracervical tampon. The fingers compress the bleeding surface and as more and more of the placental surface is demanded, either by the retraction of the womb or the separation of the placenta, the conical hand follows it, continually checking the hemorrhage, until the whole hand is in the cavity of the uterus, the forearm still continuing this pressure and occluding the os until the process is reversed by the withdrawal of the hand in the process of extraction, when the child's limb takes the place of the operator's arm. Under favorable circumstances it takes less time than any of the other procedures before mentioned, but can only come into execution when labor has progressed some considerable time. With the exception of the early tampon, it may supersede all other measures and safely terminate a desperate case that would baffle all other means.

The majority of cases in consultation that I have seen, I have delivered in this manner and, I am happy to state, with but one death and that was not due to the method, but other circumstances. In all of these labor had proceeded to such a degree, although the loss of blood had been previously very copious, that I could at once check further bleeding and rapidly and safely terminate labor. In two or three desperate cases I thus effectually tamponed the cervix until the patient could be placed under an anesthetic, and through a relaxing os I could then penetrate into the uterus without the slightest injury to the cervix.

Although there are exceptions, the cervix in placenta previa is more soft and dilatable than in ordinary conditions—it is true, frequently also exceedingly friable, requiring extreme caution in the manipulation. This by no means new method is fast finding favor with very eminent authorities and, as may be seen from the list mentioned in the beginning of this paper, is more frequently practised than any other one method. When speaking of accouchement forcé as a safe procedure, the initial manual dilatation should always be understood.

No reference has been made, so far, to separation of the placenta as a distinct method of treatment. The idea that total separation of the placenta would arrest the bleeding was based upon the erroneous theory that the placenta was the sole source of hemorrhage. This practice of Simpson and Radford has, therefore, fallen into disuse, although cases might occur in which such a course would be necessary when it would be impossible to reach the fetus for the purpose of delivery by any other means. Then it becomes necessary to follow immediately with extraction of the child, which would otherwise surely die of asphyxia and also to compress the bleeding maternal placental site in the interest of the mother.

The following case, in which this was unintentionally practised, shows the possibilities and indications of this procedure :

A few months ago I was summoned in the evening to attend a case which was reported to be one of miscarriage. The young woman was little over twenty years of age and, according to the statements of her husband, had three abortions in rapid succession and one labor at term; the child died soon after delivery. I was told that in this impending miscarriage she was in attendance by a physician, a friend of mine, who had seen the case in the morning but could not be found in the evening. The hemorrhage had been slight all day, but had become much more profuse in the evening and hence the alarm. I communicated with the attending physician (who had returned to his residence by this time) and was requested by him to visit the case for him that night and he would see it in the morning.

On my arrival at the house I found that there had been considerable hemorrhage and I prepared to tampon as for abortion. After the vagina had been plugged with iodoform gauze I found that the blood continued to oose and that the patient showed signs of syncope. I at once removed the tampon, passed my hand into the cervix and found it covered by placental tissue. As the bleeding now was becoming very profuse, I attempted to rupture the membranes, but found that I could nowhere reach the membranes, the case being one of complete placenta previa. I then endeavored to pierce the placenta through its substance with my finger, in the hope of making bipolar version. I could not succeed with this, as the placenta seemed to yield to my finger and was simply pushed up higher into the uterus, the bleeding becoming still more profuse, notwithstanding that I was making firm counterpressure upon the uterus. I found, however, that this pressure, when I withdrew my finger, momentarily checked the bleeding, and as the patient was very unruly and begged me not to proceed any further, I took advantage of this temporary respite and sent for assistance, in the meantime maintaining the position of my hands in the attitude of internal and external pressure. When my assistant arrived I had him anesthetise the woman so that I could penetrate higher upwards into the cavity of the uterus through the thick placenta. Suddenly, to my great surprise, after a hard expulsive pain, the whole placenta was thrust into my hand by the uterus, and I delivered it *en masse*. By this time the cervix was sufficiently dilated so that I could gradually introduce my hand and in a few minutes extracted the child by podalic version. The child was alive, of about six months' gestation, but died about one hour after delivery. The mother made an uninterrupted recovery without the slightest evidence of post-partum hemorrhage or rise in pulse and temperature. The cervix had not been torn in the least, and with the exception of an old laceration which had occurred in the first delivery and undoubtedly was the cause of the subsequent abortions, there was nothing abnormal in appearance. At a later time I curetted the uterus and repaired the laceration with apparent perfect restoration of the organ to its normal condition.

A somewhat similar case has been recently reported by Charles A. Whitney.[11] A multipara was suddenly seized with labor pains attended with great hemorrhage. During one of these pains the whole placenta (which had also been central) was expelled and hemorrhage ceased. As the labor pains had also ceased and the patient was much exhausted, restoratives were applied and later the child was extracted. This woman also made a good recovery.

The other methods of separating the placenta, as proposed by Barnes, Cohen, Murphy and others, chiefly applicable to lateral implantations, are, under certain conditions, valuable as auxiliary measures. They certainly cannot be regarded as a mode of treatment *per se*, for clinical experience has proven sufficiently that the hemorrhage frequently continues unabated in spite of the detachment.

For the purpose of converting a central into a lateral implantation, pushing the placenta aside in order to reach the membranes or to allow the head to descend into the os, this method in conjunction with the others previously mentioned, may often serve a useful purpose if the anatomical relations of the placenta to the cervix have been previously well studied.

Before concluding this chapter it might be of interest to describe a novel procedure by Nyhoff,[12] of Amsterdam, in the treatment of placenta previa centralis. After having made the observation that the amnion is but loosely attached to the placenta and in order to prevent the placenta from being drawn away during the process of retraction of the uterus, he proposes to pierce the placenta, strip off the amnion during the dilating period and thus enable it to follow the changes in the uterine walls. During a pain the amniotic sac bulges into the rent of the placenta, which is gradually widened until the child is born spontaneously. If the amnion is, however, very friable or too firmly adherent, he prefers delivery by the combined method with extraction of the leg. He claims for it the following advantages: (1) it does not interfere with the natural progress of labor as do rupture of the membranes and combined version; (2) it may be combined with aseptic tamponing; (3) no anesthetic is necessary; (4) it removes the temptation of rapid extraction; and hence (5) prevents cervical lacerations.

It is indicated in placenta previa centralis when the os permits the introduction of two fingers and the pains are not weak. It is contraindicated in placenta previa lateralis and when pains are absent.

SUMMARY.

*The tampon is indicated*—(1) In hemorrhage towards the end of pregnancy; (2) in the beginning of labor when os is closed; (3) in moderate dilatation of the cervix—then use cervical tampon.

*Contraindicated*—(1) When dilatation is complete or nearly complete; (2) when it fails to arrest hemorrhage even when dilatation is not far advanced.

*Rupture of the membranes indicated*—(1) When os is well dilated and either spontaneous labor or artificial delivery may occur; (2) when by this method hemorrhage is better controlled than by other means; (3) when in the absence of labor pains it will be followed by immediate pressure of the presenting part.

*Contraindicated*—(1) When os is undilated and pains good; (2) in faulty presentation of the fetus, unless it can be followed immediately by version.

*Version is indicated*—(1) When the os will admit two fingers and combined version can readily be made—Braxton Hicks's method; (2) when the os is well dilated or dilatable and hemorrhage is profuse—direct or internal version; (3) in desperate cases—accouchement forcé.

*Contraindicated*—(1) When with a moderately dilated os combined version cannot be skilfully made (the cervical tampon); (2) when with a well-dilated os, after rupture of the membranes, the head immediately engages in the cervix.

In all cases strict supervision from the onset of labor to the end of delivery.

To summarise, the following should be the treatment in the order of time in placenta previa, depending on the amount of hemorrhage and condition of the patient:

(*a*) *Before labor*—(1) Hemorrhage slight, expectant treatment; (2) hemorrhage moderate, vaginal tampon; (3) hemorrhage profuse, also try tampon and then induce labor.

(*b*) *In the beginning of labor*—(1) Hemorrhage moderate, first tampon vagina until dilatation sufficient for the introduction of the cervical bag, or, if skilled assistance be at hand, Braxton Hicks's method of bipolar version; (2) hemorrhage profuse, first cervical tampon; if not successful, gradual manual dilatation of os until

(*c*) *Labor is well in progress*—Then rupture membranes and deliver by podalic version, or if hemorrhage is arrested by the descending head, apply forceps, or if pains be good, permit spontaneous expulsion.

2. VII. Congress d. Deutschen Gesellschaft f. Gynækologie in Leipzig, 1897. Ref. Monatsch. f. Gynæk., July, 1897.
3. System of Obstetric Medicine and Surgery, p. 584.
4. Lehrbuch der Geburtshülfe, 9th ed., p. 708.
5. Ibid, p. 707.
6. Hand. d. Geburtshülfe, Vol. II., p. 76.
7. Ibid, p. 78.
8. Handbuch d. Geburtshülfe, last ed, p. 372.
9. Lomer, American Journal of Obstetrics, Vol. XVII., 1884.
10. Runge, Archiv für Geburtshülfe, Vol. XLI.
11. New York Polyclinic, 1896.
12. Monatschrift f. Geburtsh. and Gynækol., Vol. IV.

722 Laurel Street.

